# Hydromethanolic Crude Extract of the Leaf of *Urtica simensis* Hochst. ex. A. Rich. (Urticaceae) Acquires Appreciable Antiulcer Effect: Validation for *In Vivo* Antiulcer Activity

**DOI:** 10.1155/2021/6591070

**Published:** 2021-07-20

**Authors:** Woretaw Sisay, Yared Andargie, Mulugeta Molla, Alefe Norahun

**Affiliations:** ^1^Department of Pharmacy, College of Health Sciences, Debre Tabor University, Debre Tabor, Ethiopia; ^2^Department of Pharmacy, Teda Health Science College, Gondar, Ethiopia

## Abstract

**Background:**

*Urtica simensis* has been used for the treatment of peptic ulcer disease in Ethiopian folkloric medicine by drinking its juice after boiling the semicrushed leaf. To our latest understanding, no *in vivo* study was available regarding its antiulcer activity. The present study was done to appraise the ulcer-protective and ulcer healing activity of hydromethanolic crude extract of leaf of *U. simensis* in rats.

**Methods:**

Preliminary qualitative phytochemical screening and oral acute toxicity were carried out using a standard protocol. To validate *U. simensis in vivo* antiulcer potential pyloric ligature, cold restraint stress and acetic acid-induced ulcer models were employed. The extracts (100, 200, and 400 mg per kg of body weight per day), standard treatment (omeprazole 20 mg/kg/day), and vehicle (distilled water 10 ml/kg/day) were given to treatment, positive, and negative controls by oral gavage, respectively. Parameters were then evaluated accordingly after the humane scarification of rats.

**Results:**

Any sign of toxicity was not observed in the oral acute toxicity test. The crude extracts exerted a significant (*P* < 0.05) inhibition of ulcer risk compared to the negative control. In the pylorus ligation-induced ulcer model, its antisecretory activity was in a dose-dependent manner. The highest gastroprotective effect (67.68%) was exhibited by the 400 mg/kg/day dose of 80% methanolic crude extract. Regarding the chronic ulcer model, treatment at a dosage of 100, 200, and 400 mg/kg/day cures ulcers by 33.54%, 58.33%, and 67.07%, respectively, as compared to the negative control groups remarkably.

**Conclusion:**

The findings of the present study confirmed the safety and a promising *in vivo* ulcer healing and antiulcerogenic activity of *U. simensis*, thus supporting the traditional claim. In-depth investigations on the plant, however, are highly recommended.

## 1. Introduction

The gastrointestinal tract resistance from an insult occurs due to a great variety of hostile factors, including hydrochloric acid, alcohol, refluxed bile salts, and other aggressive factors. This is due to the availability of defensive and curative mechanisms for injured stomach epithelium when an insult appears [1]. Mucus barrier constituents the first line of gastric mucosal defense consisting of mucus, bicarbonate anions, and phospholipids [2].

The second line of mucosal defense is formed by an epithelial cell, which is responsible to secrete viscous mucus and HCO_3_^−^ and prostaglandins, heat shock proteins, and cathelicidins [3]. Regular renewal from mucosal progenitor cells is able to maintain the structural integrity of the gastrointestinal mucosa [4].

Peptic ulcer disease is a break in the lining of the stomach, the first part of the small intestine, or occasionally the lower esophagus due to contact with the chloridopeptic secretions [5].

About 80% of the people in the developing world depend on traditional medicine for their primary health care needs. For many decades, plants were utilized for the therapy of many diseases, PUD. Medicinal plants possessing active principles such as flavonoids, saponins, tannins, and terpenes are found to show antiulcer activity [6]. Leaf crude decoction of *Jasminum grandiflorum* [7], *Orbignya phalerata* and *Euterpe edulis* [8], *Lafoensia pacari A*. *St.-Hil* [9], *Azadirachta indicia* [10], *Carica papaya* [11], and *Plantago lanceolata L.* [12] possesses antiulcer efficacy. The efficacy and mechanisms of activity of crude herbal extracts differ in accordance with the composition of their secondary chemical metabolites [13].

*U. simensis* belongs to the kingdom Plantae, phylum Magnoliophyta, class Magnoliopsida, order Urticales, family Urticaceae, and genus *Urtica.* The family *Urticaceae* is commonly known as the nettle family which comprises a list of 48 genera and more than 2000 species of plants. Geographically, the species of these plants are mostly found in the tropical and subtropical regions of the world [14, 15].

The genus *Urtica* is used as herbal medicine to heal a variety of diseases. This family was the source of many ingredients for the treatment of PUD in the world. *U. dioica* L. has been proved to have *in vivo* antiulcer activity which might be attributed to its antimicrobial, antioxidant, anti-inflammatory, and analgesic effects [16, 17]. According to Kavalali, there are near to fifty active phytochemical compounds in the *Urtica dioica* plant including phenols and triterpenes which might be the stand for the medicinal value of the plant [18].

*U. simensis* is one of the species of nettle which is endemic in Ethiopia. It grows in the highlands of Ethiopia especially in the Amhara, Oromia, and Southern regions of Ethiopia throughout the year at 1500–3500 meters above sea level [18, 19]. It is commonly known as “Nettle” (English), “Sama” (Amharic) [20]. It is used to treat different illnesses in Ethiopia folkloric medicine. For the treatment of gonorrhea, root and leave parts of *Urtica simensis* were powdered into smaller pieces and mixed in water, and the filtrate was drunk [21]. The fresh leaves of *Urtica simensis* were also used as a well-celebrated remedy for the therapy of abdominal ache, heartburn, gastritis, and other symptoms with some assimilation in two forms of preparation. Hence, the leaves are roasted and ground into smaller pieces, and the resulting juice was taken orally or the fresh leaves could be cooked and eaten by “injera” [22]. It was declared to be used for the therapy of acute stomachache (the sap was drunk orally) and heart failure (fresh leaf steam vapor allowed to enter nasally and fumigated whole-body) [23]. In addition, the root of the plant is indicated for the therapy of plasmodial malaria infection after being squashed and shade dried and then mixed with tape water, one glass of the concentrate was drunk, and plenty of milk was drunk [24, 25]. Scientific studies have validated the ethnomedicinal claims that the leaf of *U. simensis* is useful in the management of diabetes mellitus and it has a cardioprotective activity [26, 27].

PUD affects 10% of people in the world [28]. Globally, it accounts for approximately 1% mortality. Despite the promises of the wide range of options of prescription medications, these drugs are associated with numerous side effects, which are intolerable for many patients. In addition, due to an increase in cost, drug interactions, relapse, and resistance which limit their use, this generally leads to finding out new drugs for peptic ulcer therapy [29]. The search for new and novel agents has been intensively promoted nowadays.

An ethnobotanical survey done in Ethiopia [25, 30] expressed that the leaves of *U. simensis* are given orally for 2 to 3 days for therapy of peptic ulcer diseases. To the best of our in-depth search, the latest surveys expressed that no animal experimentation data was available on the antiulcer activity of *U. simensis*. Therefore, the main aim of the present experimental study was to validate the antiulcerogenic and ulcer healing efficacy of hydromethanolic crude leaf extract of *U. simensis.*

## 2. Materials, Chemicals, Supplies, and Methods

### 2.1. Materials, Chemicals, and Supplies

The following drugs, chemicals, and instruments were used in the experiment during the study period: phenolphthalein (RFCL; RANKEM, India), absolute methanol (ReAgentchem. Ltd., India), glacial acetic acid (Lobe chemi, India), omeprazole (Cadila Pharmaceuticals, Bengaluru, India), cimetidine (ACILOC, Cadila, Ethiopia), 0.9% normal saline (Cadila Pharmaceuticals, Bengaluru, India), distilled water (EPharm, Ethiopia), ketamine (Fisher Scientific, UK), diazepam (Fisher Scientific, UK), buffered formalin (10%), sulfuric acid (Fisher Scientific, UK), sodium phosphate buffer (Hi Media Laboratories Pvt. Ltd., Mumbai, India), HCL (Nice Laboratory Reagent), 2% ferric chloride (Super Tek Chemicals), benzene (Nice Laboratory Reagent, Kerala, India), 10% ammonium hydroxide (Rankem, Mumbai, India), 0.01N sodium hydroxide (Central Drug House India), acetic anhydride and Mayer's reagent (May and Baker Ltd, England, formalin (Blulux Laboratories Pvt. Ltd., Faridaban, India), centrifuge (Hettich, Germany), automatic shaker (MaxQ 2000-USA), Whatman grade No. 1 filter paper (Schleicher and Schuell Microscience Gmbh, Germany), lyophilizer (LANCONCO–Freeze Dry System, USA), grinder (KZ-III, Wuhan, China), and rota vapor (Rotary Evaporator RE300, UK). All chemicals were analytically graded and purchased from the Ethiopian Pharmaceutical Fund Supply Agency and obtained from the University of Gondar School of Pharmacy laboratories.

### 2.2. Plant Collection and Identification

*U. simensis* leaves were gathered from Debre Tabor found in the Debub Gondar Zone of the Amhara Regional State of Ethiopia, about 655 km from the capital, Addis Ababa, at a latitude and longitude of 11°51′N 38°1′E with an elevation of 2,706 meters above sea level during January 2021.

Botanical identification of the plant was made by Dr. Getinet Masresha, Botanist, University of Gondar, Ethiopia, and the voucher specimen number Wor1/2021 was deposited at the University of Gondar herbarium for future documentation.

### 2.3. Preparation of Plant Leaf Extract

Newly collected leaves of *U. simensis* were properly irrigated with tap water to avoid dust and dried in shadow placed at room temperature. After full drying, it was crushed by a grinder (KZ-III, Wuhan, China). The dry pulverized leaves of *U. simensis* were extracted via cold maceration technique with 80% hydromethanol. One-kilogram sample was subjected to a total of 8 liters of 80% hydromethanol with four Erlenmeyer flasks and placed on an automatic shaker at 120 rpm for 72 hours. The extract was first filtered using muslin cloth followed by Whatman's filter paper no. 1. The residue was separately reexposed with the same solvent twice to maximize the yield. Then, filtrates were allowed to become concentrated in a rotary evaporator at a temperature of 40°C and 60 rpm. The filtrate was frozen overnight by treating it with a deep freezer. Then, it was lyophilized at −50°C and vacuum pressure (200 mBar) to avoid water. In the end, the percent yield of extract was analyzed, labeled, and kept in desiccators at −4°C [31]. The yield (%) was obtained from the following formula adopted from Tembe Fokunang et al. [32].

(1) $$\begin{array}{c} \text{percentage yield}=\frac{\text{mass of the crude extract obtained}}{\text{mass of the initial powder}}*100. \end{array}$$

### 2.4. Preliminary Phytochemical Analysis

Secondary plant metabolites are phytochemicals that are known to reveal therapeutic efficacy against multiple diseases such as malaria, diabetes mellitus, and peptic ulcer disease in humans and therefore might elaborate the cultural utilization of medicinal herbs for the therapy of some complications. These are phytochemical compounds with complicated structures and with more limited distribution than principal plant metabolic compounds. Secondary plant metabolic products are not vital for the plant, but till now, their physiological role is not well discovered. Standard phytochemical tests were applied to identify the major secondary plant metabolic chemicals such as polyphenols, cardiac glycosides, saponins, plant sterols, anthraquinones, flavonoids, tannins, alkaloids, and terpenoids [33, 34].

### 2.5. Acute Oral Toxicity Study

This test was applied on healthy, nonlactating, and nonpregnant female Wistar albino rats utilizing OECD, 2008 425 guidelines. Consistently, five albino rats of 8–12 weeks of age were used to approximate the LD_50_ of *U. simensis* leaf crude extract. All laboratory animals fasted (food but not water) 24 hrs before and 4 hrs after therapy with the extract. The first rat was treated with a dose of 2000 mg/kg PO to perform a sighting study. No mortality was recorded when evaluated for the first day. Then, the coming four other rats were treated similarly. They were housed individually and followed up unceasingly for 4 h with a 30 min interval and then every day for 14 days to check if there were any signs of toxicity such as behavioral profile (alertness, restlessness, irritability, and fearfulness), autonomic profiles (salivation, flow of tears, sweating, piloerection, enuresis, and defecation), neurologic (CNS) profile (spontaneous activity (drowsiness), reactivity, touch response, pain response, seizure, agitation, and tread), physical states such as anorexia, morbidity or mortality, and other signs of toxicity [5, 35].

### 2.6. Experimental Animals

Healthy adult Wistar albino rats of either sex weighing 150–250 gm inbred at the Ethiopian Health and Nutrition Research Institute (EHNRI) were selected randomly for the study. They were kept in plastic cages (6 rats per cage) at room temperature (25 ± 2°C, 12 : 12 h light and dark cycle), with water *ad libitum* under standard laboratory conditions and fed with a pellet diet (NPD). They were allowed to adapt to the laboratory environment for five days and then blindly classified into groups before each aspect of the trials. Laboratory animals were handled and cared for in the experimental study in accordance with the local ethical and internationally developed laboratory animal use, care, and welfare standard guidelines such as Basel declaration, ICLAS Ethical Guideline, and EU directive on the protection of animals used for scientific purposes [36].

### 2.7. Grouping Experimental Animals

Animals were classified into five groups consisting of six rats per group as per the models involved in the study and abstain from food in each cage for 24 hrs before the study. The rats were put with wide-mesh wire bottoms to prevent coprophagia during the experiment. The models involved were cold restraint stress and pyloric ligation-induced acute ulcer models and acetic acid-induced chronic ulcer models [12]. Negative controls were treated with distilled water (10 ml/kg), whereas positive controls were with a standard antiulcer agent (omeprazole 20 mg/kg/day in pylorus ligation-induced ulcer model or cimetidine 100 mg/kg/day in cold restraint stress-induced and acetic acid-induced ulcer models). The treatment groups received low (100 mg/kg/day), medium (200 mg/kg/day), or highest (400 mg/kg/day) dosages of crude extract. The given doses were determined based on the safety of the *U. simensis* in the acute toxicity study.

### 2.8. Pylorus Ligation-Induced Ulcer

The negative control group (category I) was pretreated with distilled water, while the positive control group (category II) was pretreated with omeprazole 20 mg/kg/day. Treatment groups (categories III, IV, and V) were pretreated with 100, 200, and 400 mg/kg/day of hydromethanolic crude extract of leaf of *U. simensis.* All study groups were pretreated for 10 consecutive days.

Before the study was started, Wistar albino rats fasted for 24 h with water *ad libitum*. An hour after the last drug therapy, animals were anesthetized with ketamine (50 mg/kg) in combination with diazepam (5 mg/kg) intraperitoneally, and by a midline incision below the xiphoid process, the abdomen was opened. The pylorus of the exposed stomach was pulled out and tied up in a tight knot with small treads at the pyloric sphincter. The abdomen was returned and closed up by using Merisilk No. 2. Four hours later, rats were euthanized by cervical dislocation. The abdomen was opened, and the cardiac end of the stomach was dissected out, and its contents were drained into a capillary tube. After treatment of gastric secretions with a centrifuge (Hettich, Germany) at 2000 rpm for 10 minutes, the extent of the supernatant was recorded and taken for the quantification of full acidity and pH. The gastric mucosa was subjected to normal saline and distilled water, labeled, and immersed in sodium phosphate-buffered 10% formalin until it was evaluated for ulceration by using a hand lens (10x) consistently.

### 2.9. Cold Restraint Stress-Induced Ulcer

The hypothermic restraint stress-induced gastric ulcer model was evaluated on Wistar albino rats by the mechanism designed by Levine with some modifications [37]. Animals were categorized into five classes comprising six animals each. The animals fasted for 24 h prior to the experiment.

The negative control group (category I) were pretreated with distilled water (10 ml/kg/day) while the positive control group (category II) were with cimetidine (100 mg/kg/day). Treatment groups (categories III, IV, and V) were pretreated with 100, 200, and 400 mg/kg/day of hydromethanolic crude extract of leaf of *U. simensis.* All study groups were pretreated with a single oral dose.

An hour after therapy, animals were immobilized inside a closed cylindrical cage and kept inside the refrigerator which was adjusted to 2–4°C in order to induce gastric ulceration. Three hours later, animals were euthanized by cervical dislocation, the stomach was detached and unfold along the greater curvature. Then, it was gently washed with water to avoid the gastric contaminants and blood clots and inspected and evaluated for ulcer lesion [38, 39].

### 2.10. Acetic Acid-Induced Chronic Ulcer

The model designed to be used was that of Takagi et al. with some rearrangements [40]. Animals fasted for 24 h with water *ad libitum*. During the actual experiment, the rats were anesthetized with 50 mg/kg of ketamine in combination with 5 mg/kg of diazepam intraperitoneally. Upon laparotomic technique, the abdominal wall was unfolded and treated with 20% glacial acetic acid (50 *μ*l) injection into the subserosal layer in the glandular part of the anterior wall for the sake of gastric ulcer induction. The stomach wall was cleaned with water in order to avoid adhesion to the outer surface of the ulcerated region. The abdomen wall was then closed by interrupted sutures using surgical chromic catgut No. 2/0 and Merisilk number 2.

A day later to surgical procedure, each study group was treated accordingly. The negative control group (category I) were treated with distilled water (10 ml/kg/day). The positive control group (category II) was treated with cimetidine (100 mg/kg/day). Treatment groups (categories III, IV, and V) were treated with 100, 200, and 400 mg/kg/day of hydromethanolic crude extract of *U. simensis.* All study groups were treated for twenty consecutive days. Animals were euthanized by cervical dislocation by the 21^st^ day, the stomach was removed, and also the mucosal injury was assessed [9].

### 2.11. Parameters for Evaluation of Antiulcer Activity

#### 2.11.1. Macroscopic Evaluation of Stomach

In order to evaluate the ulcer formations, the abdomen was unfolded along the greater curvature, cleaned with normal saline to strip stomach contents and clots, and evaluated by a 10x magnifier hand lens. After the numbers of ulcerations would be counted, the scoring of the ulcer was made as follows based on the method designed by Kulkarni SK [41]: normal colored GI mucosa (0), red-colored stomach mucosa (0.5), external mucosal ulcerations (spot ulcer) (1), hemorrhagic line (1.5), in-depth ulcerations (2), and perforation/penetration of stomach (3). Ulcer index (UI) was calculated using the following formula:

(2) $$\begin{array}{c} \text{UI}=\frac{\text{UN}+\text{US}+\text{UP}}{10}, \end{array}$$

where UI is the ulcer index; UN is the average number of ulcers per laboratory animal; US is the average number of severity scores; UP is the percentage of animals contracted with mucosal ulceration.

Percentage inhibition of ulceration was calculated as follows:

(3) $$\begin{array}{c} \text{\% inhibition of ulceration}=\frac{\text{UI control}-\text{UI test}}{\text{UI control}}\times 100,\\ \text{\% protective ratio}=100-\left(\frac{\text{UI pretreated}}{\text{UI control}}\right)\times 100,\\ \text{\% curative ratio}=100-\left(\frac{\text{UI treated}}{\text{UI control}}\right)\times 100. \end{array}$$

#### 2.11.2. Determination of pH and Volume of Gastric Secretions

The gastric content collected in a glass tube is used to measure both the volume and pH of the gastric juice after centrifugation at 2000 rpm for 10 minutes. In order to determine the pH of gastric secretions, 1 ml of each aliquot from the supernatant was utilized.

#### 2.11.3. Evaluation of Full Acidity

A portion of 1 ml gastric juice mixed with 9 ml of distilled water was added to a 50 ml round flask, and two drops of phenolphthalein indicator was replaced to it and titrated with 0.01N sodium hydroxide until a permanent pink color appeared. The volume of 0.01N sodium hydroxide utilized was recorded. The full acidity is revealed by SI unit mEq/L by the following method [42]:

(4) $$\begin{array}{c} \text{full acidity}=\frac{\text{V NaOH}\times \text{N}\times 100\text{mEqlL}}{0.1}. \end{array}$$

### 2.12. Data Quality Assurance

Data quality assurance was maintained by categorizing experimental laboratory animals by a random sampling statistical method, collecting data of all indicators blindly, maintaining and applying standard procedures consistently, and using scientifically labeled instruments.

### 2.13. Data Management and Analysis

The collected data were organized, entered, and analyzed using SPSS version 24, and outcomes were revealed as mean ± SEM. One-way analysis of variance (ANOVA) followed by post hoc Tukey's HSD multiple comparison test was utilized for comparison between control and treatment groups. *P* values less than 0.05 at a 95% confidence interval were regarded as statistical significance.

## 3. Result

### 3.1. Percentage Yield Determination of the Crude Extract

One hundred and forty-nine grams of hydromethanolic crude extract was obtained from 1 kg leaf of *U. simensis* (see Table 1).

### 3.2. Preliminary Phytochemical Screening

These qualitative tests were done in accordance with standard test guidelines. These tests revealed the existence of terpenoids, saponins, tannins, flavonoids, alkaloids, and phenolic compounds (See Table 2).

### 3.3. Acute Oral Toxicity Study

This test was done in female albino rats based on OECD-425 guidelines (OECD, 2008). Systemic toxicities such as behavioral profiles (attentiveness, agitation, resentment, and dread), autonomic profiles (froth, lacrimation, sweating, piloerection, enuresis, and excretion), neurologic (CNS) profile (spontaneous activity (drowsiness), nervousness, tactile response, ache response, seizure, trembling and pace), physical states such as anorexia, morbidness or lethality, and others were not revealed from female albino rats involved. Upon this outcome, we can say that the mean lethal dose of the candidate plant extract (LD50) was beyond 2000 mg/kg.

### 3.4. Antiulcer Activity in Relation to *U. simensis*

#### 3.4.1. Antiulcer Activity on Pylorus Ligation-Induced Ulcer in Rats

Pylorus ligation resulted in the accumulation of gastric secretions of 4.73 ± 0.17 ml with pH 1.76 ± 0.16 and total acidity of 57.16 ± 0.93 mEq/L in the negative control group. Pretreatment significantly brings down the volume of GI secretions to 3.72 ± 0.23 (*P* < 0.01), 2.79 ± 0.25 (*P* < 0.001), and 1.93 ± 0.05 (*P* < 0.001) ml at the respective doses of 100, 200, and 400 mg/kg/day. pH was significantly (*P* < 0.001) increased to 4.67 ± 0.26, 5.79 ± 0.12, and 6.09 ± 0.099 at an ascending order of respective doses. Besides, full acidity was shrunk remarkably (*P* < 0.001) in comparison with negative control groups (see Table 3). Ulcer protection at a dose of 400 mg/kg/day of test extract was equivalent to that of the standard (see Table 4).

**Figure 1:**
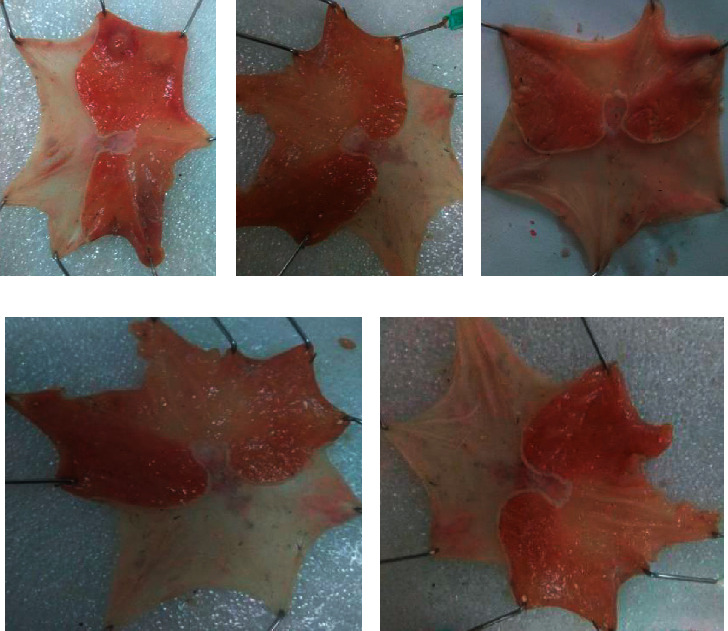
Pyloric ligation-induced gastric ulcer in rats. (a) Negative control treated with distilled water 10 ml/kg/day; (b) negative control treated with 100 mg/kg/day crude extract; (c) negative control treated with 200 mg/kg/day crude extract; (d) negative control treated with 400 mg/kg/day crude extract. (e) Positive control treated with standard (omeprazole 20 mg/kg/day). All were pretreated for 10 consecutive days.

Furthermore, it was observed that pyloric ligation has caused gastric ulceration, and pretreatment with the crude extract has lowered gastric ulceration in a dose-dependent manner (see Figure 1).

#### 3.4.2. Effect on Cold Restraint Stress-Induced Ulcer

The crude extract reduced ulcer score markedly to 18.75 ± 1.01 (*P* < 0.001), 12.42 ± 2.60 (*P* < 0.001), and 7.50 ± 1.59 (*P* < 0.001) at a respective dose of 100, 200, and 400 mg/kg/day.

**Figure 2:**
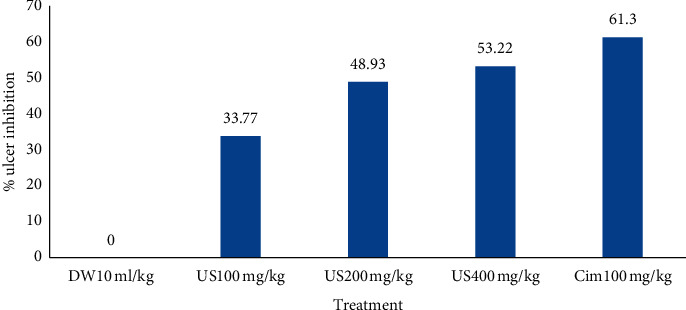
Percentage ulcer inhibition effect of crude extract on cold restraint stress-induced ulcers in rats. DW10 ml/kg: distilled water 10 ml/kg/day, Cim100: cimetidine 100 mg/kg/day, US100 mg/kg: *U. simensis* 100 mg/kg/day, US200 mg/kg: *U. simensis* 200 mg/kg/day, US400 mg/kg: *U. simensis* 400 mg/kg/day.

It also reduces ulcer index at a statistically significant level only at 200 and 400 mg/kg in comparison with negative controls (see Table 5). Percentage reduction of the ulceration was 33.77%, 48.93%, and 53.22% in animals pretreated with 100, 200, and 400 mg/kg/day of *U. simensis* and 61.30% in those treated with the standard (see Figure 2).

**Figure 3:**
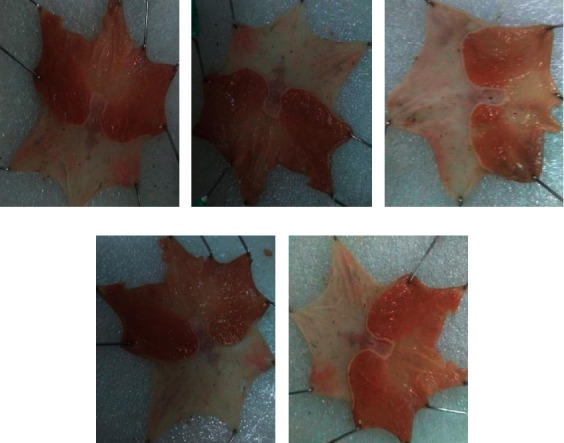
Cold restraint stress-induced ulcers in rats. (a) Negative control treated with distilled water 10 ml/kg/day); (b) negative control treated with 100 mg/kg/day crude extract; (c) negative control treated with 200 mg/kg/day crude extract; (d) negative control treated with 400 mg/kg/day crude extract. (e) Positive control treated with the standard (cimetidine 100 mg/kg/day). All were pretreated with only a single dose.

A spot ulcer and many hemorrhagic streaks were observed in the negative control while some hemorrhagic streaks and a minimal number of spots were also observed in the lower dose of the extract. As we go in depth from lower to higher doses, it showed that significant ulcer formations were not observed (see Figure 3).

#### 3.4.3. Effect on Acetic Acid-Induced Ulcer

**Figure 4:**
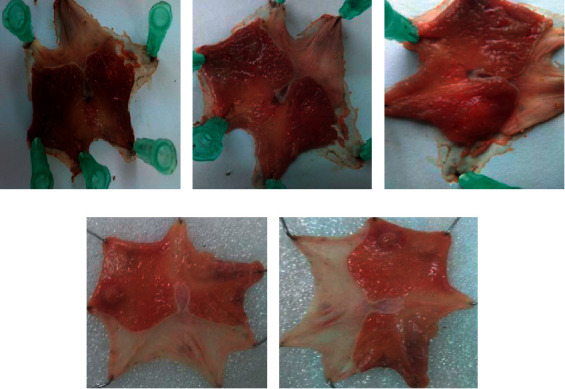
Acetic acid-induced gastric ulcers in rats. (a) Negative control treated with distilled water 10 ml/kg/day; (b) negative control treated with *U. simensis* 100 mg/kg/day crude extract; (c) negative control treated with *U. simensis* 200 mg/kg/day crud extract; (d) negative control treated with *U. simensis* 400 mg/kg/day crud extract. (e) Positive control treated with standard cimetidine 100 mg/kg/day. All were treated for 20 consecutive days.

Crude extract cured gastric mucosal ulcerations at a rate of 33.54%, 58.33%, and 67.07% at a respective dosage of 100 (*P* < 0.05), 200 (*P* < 0.001), and 400 (*P* < 0.001) mg/kg/day twenty days of therapy later. Every dose of the crude extract can diminish the grade of gastric ulceration markedly (*P* < 0.001) in comparison with the negative controls (See Table 6). It had also a curative potential in both ulcer surface area and ulcer depth (see Figure 4).

## 4. Discussion

Peptic ulcers have unquestionably been a disease of the twentieth century. It remains part of the major challenges for gastroenterologists. Up to date, there is no conventional medicine that discharges every standardized set of therapeutic goals of peptic ulcer disease. Drug insensitivity of peptic ulcer disease has been reported, despite pharmacotherapy. Moreover, conventional drugs have been affiliated with serious unwanted outcomes; today, having novel and efficacious ulcer protective and ulcer healing agents with enhanced safety profiles is a must [43].

In the present study, the percentage yield of *U. simensis* crude extract was 14.9% which is comparable to the 13.75% yield of the previous study [44]. The findings of the present phytochemical screening tests were in line with a study at Addis Ababa University in which plant steroids were noted, but not in this work. The discrepancy may be related to seasonal or environmental differences at which the plant was collected. The oral acute toxicity study exhibited the fact that crude extract was safe in rats at a limited dose of 2000 mg/kg and that the LD_50_ of the extract is beyond the specified dose which is in line with other studies [26, 27].

First, the antiulcerogenic activity of the crude extract was assessed in the pylorus ligation and hypothermic restraint stress-inflicted acute gastric ulcer frames, whereas ulcer healing activity was in the glacial acetic acid-induced chronic ulcer model which is among the most frequently used models for screening substances with a potential of antiulcer activity [45].

Crude extracts at all doses diminished the volume and full acidity of gastric secretions and rise pH remarkably. The risk of pyloric ligation associated gastric ulcerations has been diminished significantly. The ulcer protection employed by the highest crude test extract was comparable to that of the standard. This outcome, moreover, indicates that test crude extract had a dose-dependent efficacy in averting stomach lesions in both pylorus ligation and cold restraint stress-induced ulcers in agreement with other studies [46, 47].

The corresponding gastroprotective and ulcer curative effect of *U. simensis* revealed in the present work is accredited to the chemistry of the extract. This activity is similar to other plants found in the same species, *Urtica dioica* [48]. Some phytoconstituents extracted from medicinal plants possess the antiulcerogenic activity and act by various mechanisms. Antiulcerogenic terpenoid phytochemicals include triterpenes, diterpenes, and terpenic derivatives. Carbenoxolone is a triterpene derivative, an efficient stimulator of gastroprotective mucus synthesis, enables prostaglandin synthesis at high levels, and reduces pepsin production [49, 50]. Polyphenols possess protective and therapeutic potential in peptic ulcer mediated by improving cytoprotection, reepithelialization, and angiogenesis, upregulating tissue growth factors and prostaglandins; enhancing endothelial nitric oxide synthase-derived NO; and suppressing oxidative mucosal damage and by antacid and antisecretory activity. In addition, the anti-inflammatory activity due to the downregulation of proinflammatory cytokines has a key role in the antiulcer action of polyphenols [51]. Among those polyphenols, flavonoids have antisecretory, antihistaminic, anti-*H. pylori*, anti-inflammatory, antioxidant, and gastroprotective effect, acting in the prevention of gastric lesions induced by different ulcerogenic agents [52].

Saponins have a gastroprotective activity due to antisecretory and cytoprotective effects. Aescin is a mixture of saponins that possess antiulcer activity in various ulcer models (cold restraint stress and pylorus-ligated), due to its antisecretory effect. Anisodamine and anisodine are analogs of the atropine alkaloid equipped with gastroprotective activity while they were evaluated in rats by indomethacin, stress, pylorus ligature, acetic acid, or absolute ethanol-induced ulcer models. Therefore, *U. simensis* gastroprotective and ulcer healing activity may be accounted from its saponins and alkaloids [53]. The ulcer protective activity of tannins might be associated with its ability to “tanning” and vasoconstriction effects. Their astringent action can help to precipitate microproteins on the ulcer surface, thereby forming an impermeable layer over the lining that hinders gut secretions and protects the underlying mucosa from toxins and other irritants [54, 55].

Clod restraint stress has been one of the most popular stressors in experimental medicine. It elicits the purest form of psychological frustration accompanied by vigorous struggling which means muscular exercise [56]. Cold restraint stress-inflicted acute stomach ulcer model provides both psychological and biological challenges on rats which are believed to imitate actual human patients with acute stomach wall ulcerations. This type of ulcer may come into sight in the stomach wall as a result of critical injury, surgical manipulations, or septic shock, being commonly received for evaluating the mechanics of stress-induced gastric ulcerations. This model has a central nervous system factor role in introducing PUD [57]. In this study, crude extract at medium and highest doses is able to shrink ulcer risk remarkably but not at the lowest one. The ulcer index produced in the negative control group (20.05 ± 0.36) is nearly consistent with other studies (24 ± 3.24, 19.75 ± 0.10) [39, 47] which implicates the quality of the undertaken procedure. Even though effective therapy remains elusive in the treatment of stress-induced peptic ulcers, nonantiulcer agents such as benzodiazepines and tricyclic antidepressants are known to partially or completely prevent this ulcer. Crude extract of *U. simensis* at 400 mg/kg reduces gastric ulcerations associated with cold restraint stress in rats at a rate (53.22%) comparable with ethanol extract of stem bark of *Careya arborea* Roxb. at a dosage of 300 mg/k (50.21%) [58]. The preventive capacity of *U. simensis* against tension-inflicted gastric ulcerations might be due to the antidepressant activity of its alkaloids in addition to other mechanisms that safeguard the stomach epithelium [59].

In addition to elucidating its gastroprotective effect, this experimental study as well appraises its impact on curing persistent stomach lesions. The outcomes of the present study were equivalent to the other similar studies; for example, *Jasminum grandiflorum* 80% methanolic leaf extract on a glacial acetic acid-induced chronic ulcer in rats produces an ulcer healing ratio of 56% and 66% [7]. Every dose of the crude extract can diminish ulcer grade markedly at an impact level of *P* < 0.001 in comparison to the negative controls. The lowest dosage had a remarkable discrepancy to the conventional, medium, and highest research groups which indicates that the plant had a dose-dependent ulcer curative potential.

Red ginseng-derived saponins revealed gastric lesion curing and new blood vessel production by enhancing vasoendothelial growth factor production [60]. Therefore, the persistent stomach lesion curing property of *U. simensis* also might benefit from its capacity to upregulate these factors due to its phytoconstituents such as saponins.

## 5. Conclusion

The finding revealed that the extract has a promising antiulcer activity on pyloric ligation, cold-restraint stress-induced acute, and acetic acid-induced chronic ulcer models, which corroborates its cultural therapeutic role. Upon further isolation of active phytochemicals and confirmatory tests, it might serve as a base to the discovery of novel antiulcer agents. But due to species differences, direct generalization of the outcomes of this evaluation to human beings is generally impossible.

## 6. Recommendation

This study established the plant's therapeutic value in gastric ulcerations which is reported in Ethiopian cultural therapeutic practices. In order to determine the active principle/s responsible for its antiulcer activity and to describe the exact mechanics, it requires a thorough investigation. The finding should be upheld by other relevant models that should impose gastric injury including alcohol and NSAID-induced ulcer models.

## Figures and Tables

**Table 1:** Describing quantity and quality of hydromethanolic crude extract.

Solvent	Color and texture of extract	Outcome (gm)	Outcome in percentage (W/W)

80% hydromethanol	Dark blue powder	149	14.9

**Table 2:** Qualitative phytochemical analysis of hydromethanolic crude extract.

Phytochemical	Type of tests performed	Result
Alkaloids	Wagner's test	+
Anthraquinones	Borntrager's test	−
Flavonoids	Alkaline reagent (NaOH) test	+
Glycosides	Keller–Killiani test	−
Phenolic compounds	Ferric chloride test	+
Plant steroids	Liebermann–Burchardt test	−
Saponins	Froth test	+
Tannins	Braemer's test	+
Terpenoids	Salkowski's test	+

+: exists, −: does not exist).

**Table 3:** The effect of *Urtica simensis* on gastric secretion, full acidity, and pH in pylorus-ligated rats.

Class	Therapy	Dosage	GI secretion (ml)	Depletion (%)	Full acidity (mEq/L)	Depletion (%)	PH
I	Distilled H_2_O	10 ml/kg/day	4.73 ± 0.17	0.00	57.16 ± 0.93	0.00	1.76 ± 0.16
II	Omeprazole	20 mg/kg/day	2.07 ± 0.07^w3^	56.24	17.02 ± 0.42^w3^	70.22	6.09 ± 0.17^w3^
III	*U. simensis*	100 mg/kg/day	3.72 ± 0.23^w2^	21.35	19.49 ± 0.38^w3^	65.90	4.67 ± 0.26^w3^
IV	*U. simensis*	200 mg/kg/day	2.79 ± 0.25^w3^	41.01	17.69 ± 0.38^w3^	69.05	5.79 ± 0.12^w3^
V	*U. simensis*	400 mg/kg/day	1.93 ± 0.05^w3^	59.20	16.73 ± 0.15^w3^	70.73	6.09 ± 0.099^w3^

*Note.* Each value represents mean ± SEM; *n* = 6; ^w^: against class I; ^2^: *P* < 0.01, ^3^: *P* < 0.001; SEM: standard error of the mean.

**Table 4:** The effect of *Urtica simensis* on ulcer score and ulcer index in pylorus-ligated rats.

Class	Treatment	Dose	US	% change US	UI	% change UI
I	Distilled H_2_O	10 ml/kg	81.75 ± 4.36	0.00	23.58 ± 0.68	0.00
II	Omeprazole	20 mg/kg	7.08 ± 2.39^w3^	91.34	7.71 ± 2.44^w3^	67.30
III	*U. simensis*	100 mg/kg	11.92 ± 0.82^w3^	85.42	11.71 ± 0.13^w2^	50.34
IV	*U. simensis*	200 mg/kg	7.75 ± 1.77^w3^	90.52	9.46 ± 1.20^w3^	59.88
V	*U. simensis*	400 mg/kg	6.67 ± 2.25^w3^	91.84	7.62 ± 2.41^w3^	67.68

*Note.* Each value represents mean ± SEM; *n* = 6; ^w^: against class I; ^2^: *P* < 0.01, ^3^: *P* < 0.001.

**Table 5:** The effect of *Urtica simensis* on ulcer score and ulcer index in cold restraint stress-induced ulcerated rats.

Class	Treatment	Dose	Ulcer score	Ulcer index
I	Distilled H_2_O	10 ml/kg/day	63.17 ± 2.26	20.05 ± 0.36
II	Cimetidine	100 mg/kg/day	7.25 ± 2.44^w3^	7.76 ± 2.46^w3^
III	*U. simensis*	100 mg/kg/day	18.75 ± 1.01^w3x2z2^	13.28 ± 0.14
IV	*U. simensis*	200 mg/kg/day	12.42 ± 2.60^w3^	10.24 ± 2.05^w2^
V	*U. simensis*	400 mg/kg/day	7.50 ± 1.59^w3^	9.38 ± 1.88^w2^

*Note.* Each value expresses mean ± SEM; *n* = 6; ^w^: against class I; ^x^: against class II; ^z^: against class V; *P* < 0.05, ^2^: *P* < 0.01, ^3^: *P* < 0.001; SEM: standard error of the mean.

**Table 6:** Effects of ulcer score and ulcer index on an acetic acid-induced ulcer in rats.

Class	Treatment	Dose	Ulcer score	% change US	Ulcer index	% change UI
I	Distilled H_2_O	10 ml/kg/day	82.92 ± 1.63	0.00	22.99 ± 0.27	0.00
II	Cimetidine	100 mg/kg/day	10.67 ± 3.40^w3^	87.13	8.20 ± 2.59^w3^	64.33
III	*U. simensis*	100 mg/kg/day	34.42 ± 1.47^w3x3y3z3^	58.49	15.31 ± 0.37^w1z1^	33.54
IV	*U. simensis*	200 mg/kg/day	11.00 ± 2.51^w3^	86.73	9.58 ± 1.92^w3^	58.33
V	*U. simensis*	400 mg/kg/day	6.33 ± 2.02^w3^	92.34	7.57 ± 2.39^w3^	67.07

*Note.* Each value indicates mean ± SEM; *n* = 6; ^w^: against class I; ^x^: against class II; ^y^: against class IV; ^z^: against class V; ^1^: *P* < 0.05, ^2^: *P* < 0.01, ^3^: *P* < 0.001; SEM: standard error of the mean.

## Data Availability

The datasets analyzed during the current study are available from the corresponding author on reasonable request.

## Abbreviations

ANOVA: Analysis of variance
EHNRI: Ethiopian Health and Nutrition Research Institute
ICLAS: International Council for Laboratory Animal Science
LD_50_: Median lethal dose
PUD: Peptic ulcer disease
UI: Ulcer index.
